# Automated segmentation of the mandibular canal and its anterior loop by deep learning

**DOI:** 10.1038/s41598-023-37798-3

**Published:** 2023-07-04

**Authors:** Nicolly Oliveira-Santos, Reinhilde Jacobs, Fernando Fortes Picoli, Pierre Lahoud, Liselot Niclaes, Francisco Carlos Groppo

**Affiliations:** 1 OMFS IMPATH Research Group, Department of Imaging and Pathology, KU Leuven and University Hospitals Leuven, UZ Campus St Rafael, Leuven, Belgium; 2 Department of Oral Diagnosis, Piracicaba Dental School, University of Campinas (UNICAMP), Piracicaba, São Paulo Brazil; 3 Department of Dental Medicine, Karolinska Institutet, Stockholm, Sweden; 4 Department of Stomatology and Oral Radiology, Dental School, Federal University of Goiás, Goiânia, Goiás Brazil; 5 Department of Biosciences, Piracicaba Dental School, University of Campinas (UNICAMP), Piracicaba, São Paulo Brazil

**Keywords:** Oral anatomy, Machine learning

## Abstract

Accurate mandibular canal (MC) detection is crucial to avoid nerve injury during surgical procedures. Moreover, the anatomic complexity of the interforaminal region requires a precise delineation of anatomical variations such as the anterior loop (AL). Therefore, CBCT-based presurgical planning is recommended, even though anatomical variations and lack of MC cortication make canal delineation challenging. To overcome these limitations, artificial intelligence (AI) may aid presurgical MC delineation. In the present study, we aim to train and validate an AI-driven tool capable of performing accurate segmentation of the MC even in the presence of anatomical variation such as AL. Results achieved high accuracy metrics, with 0.997 of global accuracy for both MC with and without AL. The anterior and middle sections of the MC, where most surgical interventions are performed, presented the most accurate segmentation compared to the posterior section. The AI-driven tool provided accurate segmentation of the mandibular canal, even in the presence of anatomical variation such as an anterior loop. Thus, the presently validated dedicated AI tool may aid clinicians in automating the segmentation of neurovascular canals and their anatomical variations. It may significantly contribute to presurgical planning for dental implant placement, especially in the interforaminal region.

## Introduction

The mandibular canal (MC) extends bilaterally from the mandibular foramen to the mental foramen carrying the inferior alveolar nerve (IAN) and blood vessels, innervating and vascularizing the ipsilateral lower teeth and periodontal tissues directly or through its branches. The MC crosses the mental foramen towards the interforaminal region, where it separates into the mental canal and the incisive canal^1^. While the incisive canal emerges anteriorly, the mental canal curves upward, backward, and lateral to reach the mental foramen^2^. The mental foramen location varies from below the mandibular canine to the first molar but is commonly situated below the mandibular second premolar^3,4^. Occasionally, the mental canal curving occurs more anteriorly, then looping backward and upward, forming the so-called anterior loop (AL) of the IAN^5,6^. Anatomical variations of the MC, such as AL, must be detected to avoid nerve injury during oral and maxillofacial surgical procedures, such as genioplasty and dental implant placement^6–11^.

The reported prevalence of AL ranges widely from 0 to 94% depending on geographical variations, the applied definition, and related assessment methods^3,5,10,12–14^, thus hampering comparative studies. While some studies define AL as an extension without minimal distance from the mental foramen^15^, other authors suggest that this anterior extension must be at least 1 mm or 2 mm^3,6^. Also, there seems to be a relationship between the location of the mental foramen and the presence of the AL. The AL is often detected when the mental foramen is located apically to the second premolar^12^. Clinicians may be assisted by artificial intelligence (AI) to allow for automated visualization of the entire mandibular canal trajectory. Nowadays, AI tools are increasingly introduced for detecting and segmenting anatomical structures and pathology^16–18^. Considering the importance of accurately localizing the MC anatomy, deep learning (DL) techniques were developed to automatically detect and segment the MC using cone-beam computed tomography (CBCT) images. These canal segmentation tools allow highly accurate results, highlighting the importance of DL to efficient treatment planning, especially to implant placement^19–21^. However, there is still a need to generalize the DL tools to enable the detection and segmentation of anatomical MC variations. It undoubtedly also applies to the automated detection and segmentation of AL. Therefore, the aim of the present study was to train and validate a dedicated cloud-based AI-driven tool to allow accurate and timely segmentation of the MC and its AL on CBCT scans.

## Results

Table 1 shows the mean and standard deviation of the IoU, DSC, HD, Precision, Recall, and Accuracy measures of the testing dataset. No significant difference in the metrics between the segmentation of the MC with and without AL was noted (p > 0.05). Notwithstanding, the mean of Recall and Precision metrics were 0.965 and 0.672, respectively, showing that there was more under-segmentation, meanwhile providing a global accuracy of 0.997.

**Table 1:** Mean (standard deviation) of the AI segmentation metrics of the mandibular canals with and without anterior loop (control).

Mandibular canals	AI segmentation metrics					
	IoU	DSC	Precision	Recall	Accuracy	HDmm95
Anterior loop	0.659 (0.076)	0.792 (0.053)	0.677 (0.075)	0.961 (0.035)	0.998 (0.001)	0.428 (0.102)
Control	0.654 (0.057)	0.789 (0.044)	0.668 (0.061)	0.970 (0.026)	0.997 (0.001)	0.429 (0.039)

p > 0.05.

**Figure 1:**
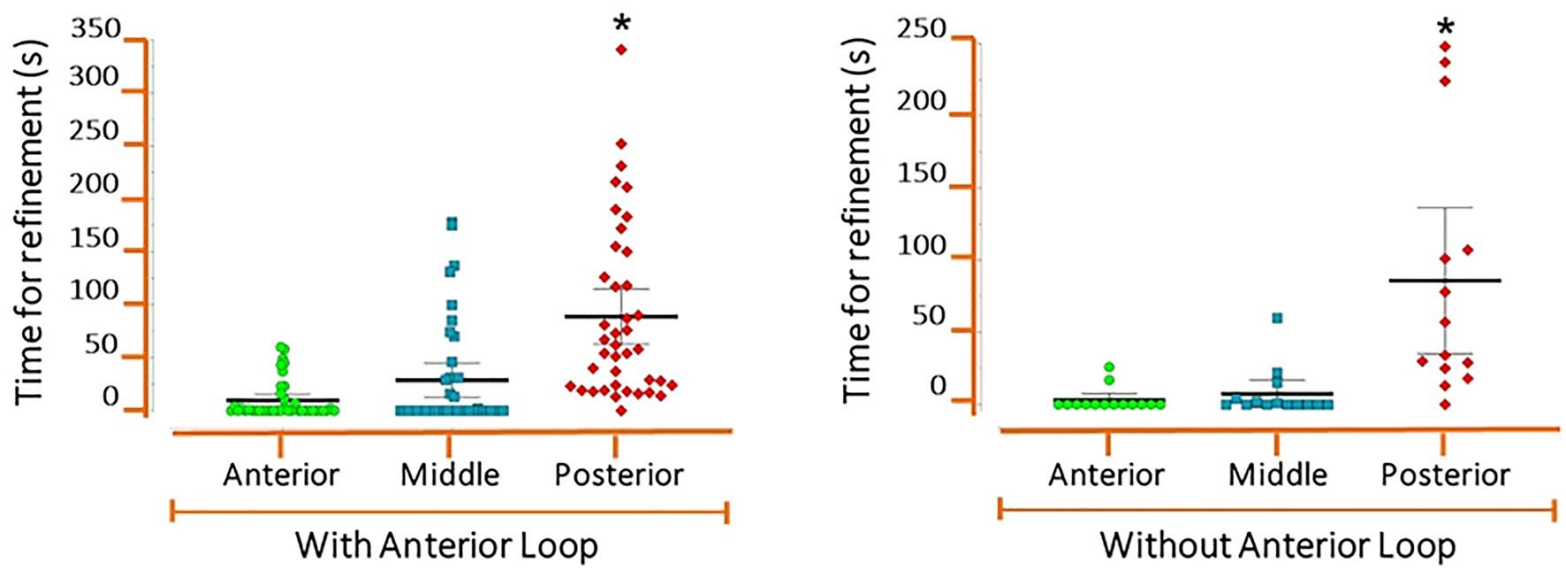
Refined AI segmentation time for the three sections. Each point represents a value. Bars = median, Swiss = interquartile deviation. *Significantly different than other sections.

The median time from AI-driven segmentation was 22.5 s, whereas the median time from refined AI segmentation was 156 s. The time for refined AI segmentation in the posterior section was longer than for the anterior and middle sections (p ≤ 0.05). There were no significant differences in the time needed to refine AI segmentation of anterior and middle sections, regardless of the presence of AL (p > 0.05) (Fig. 1). It implies that in the mental foramen region, the presence of the AL did not compromise AI segmentation. The anterior and middle sections also presented the most accurate AI segmentation.

## Discussion

The correct detection of the MC is crucial to avoid per-operative nerve injury. However, in cases of anatomical variations of the MC, such as AL, correct identification becomes a challenge, even for oral radiologists and surgeons. In such cases, the detection by AI may overcome the limitation of the human eyes. In our study, besides the MC segmentation achieving much better metrics than previous AI-based segmentation of the MC on CBCT images^19–21^, the present study is the first to show a unique AI network detecting anatomical variations such as the AL. On top of that, the currently described approach is based on Lahoud et al.^21^ being the only approach to detect yet fully, timely, and accurately segment the neurovascular canal along its course in the mandible.

Due to the anatomic complexity of the mental foramen region, such as the presence of AL, many authors highlighted the importance of the preoperative diagnosis with CBCT images during surgical planning near the mental foramen^3,6,9,14,22^. Even though there is high variability in the prevalence, length, gender, and side distribution of AL in various populations considering a high heterogeneity of reported methodologies to evaluate AL^12^. Because of this variability, some authors stated it is difficult to recommend reliable fixed safety margins for surgical procedures such as implant placement^22,23^. Therefore, following the methodology of Krishnan et al.^3^, we only considered AL if it was bigger than 1 mm, also considering that a more considerable variability from the regular course of the MC would bring a more significant challenge for the AI network to recognize. Notwithstanding, there was no difference in the AI-driven segmentation between the MC with and without AL, demonstrating that AI may standardize the evaluation of this anatomical variation and aid surgical planning of implant placement in the mental region.

More under-segmentation in the posterior section of the MC was seen compared to the middle and anterior sections. To evaluate the segmentation of the MC, Agbaje et al.^24^ also divided the MC into three regions: mental, body, and angle. They observed more under-segmentation of the MC in the angle region than in the body region; besides, the mental region presented a smaller thickness than the other regions^24^. These results indicate that the posterior section is the more challenging region to correctly segment the MC. The 3D U-Net network is based on localization and sparse annotation slices. This network allows good results with little training since data augmentation is applied to learn image invariance, which is a challenge for AI^25,26^. However, as the posterior section presents high variability of position and morphology, especially close to the mandibular foramen^27,28^, the difference found between the sections in our study may be related to this higher variability in the posterior section. On the other hand, causes of IAN injury are local anesthetic injections, endodontics, ablative surgery, trauma, orthognathic surgery, third molar surgery, and, especially, implant surgery^29^, most of them related to the anterior and middle sections, where are located the mandibular teeth. Thus, the anterior and middle sections of the MC are the most relevant sections to be correctly segmented and, consequently, avoid nerve injury.

In the present study, AI validation metrics achieved a mean recall value of 0.965, showing the almost perfect specificity of the AI network. It implies that there is little over-segmentation. Conversely, the mean precision value was 0.672, explained by the under-segmentation in the posterior section, which decreased the IoU values. Despite that, accuracy values showed an overall almost perfect segmentation. As the accuracy metrics are calculated based on the entire MC segmentation and sections, if the posterior section, which is less relevant, were not segmented and, thus, not considered in the calculation, our metrics would be even higher.

Lahoud et al.^21^ observed that the algorithm performed better in CBCTs with a higher cortication degree^21^. Since the visibility of the MC may change according to the resolution of the CBCT acquisition protocol^30^, in the present study, different CBCT machines and resolution protocols were used. Different resolution protocols increased the generalizability of the presently validated tool, achieving a global accuracy of 0.997 for both MC with and without AL. Furthermore, it indicates that our AI-driven tool allows good segmentation, regardless of the cortication of the MC. Thus, it can significantly contribute to help clinicians to correctly detect the MC in complex cases where is more challenging to distinguish it from the trabecular bone.

In the future, we will strive to obtain more generalizability of the cloud-based AI tool by training more CBCT scans from different machines and more patients with anatomical variations and variable dental status, such as pathological jaw lesions in contact with the MC. It would then allow the cloud-based AI tool to predict the performance of the network for all conditions and applications, such as for presurgical planning.

From the present validation study, the AI-driven tool provided accurate segmentation of the mandibular canal, even in the presence of anatomical variations such as an anterior loop. The presently validated dedicated AI tool may aid clinicians to automate the segmentation of neurovascular canals and their anatomical variations. This tool may help surgeons during surgical planning, such as implant placement, especially in the interforaminal region.

## Methods

The study was approved by the Ethical Committee Research UZ/KU Leuven (protocol S66447) and performed following the Declaration of Helsinki. In addition, informed consent was obtained from all participants. Lahoud et al.^21^ previously trained and validated the current AI network, as follows^21^.

### AI network development

CBCT scans were randomly collected from the local database. The CBCT scans were from dentate patients (mean age 25 ± 11 years old) that acquired the images for oral surgical purposes. The CBCT devices used to acquire the scans were NewTom VGI EVO (QR Verona, Cefla, Verona, Italy), ProMax 3D MAX (Planmeca, Helsinki, Finland), Accuitomo 170 (Morita, Kyoto, Japan), and Scanora 3Dx (Soredex, Tuusula, Finland). In addition, to increase the robustness of the AI network, CBCT scans varying field of view (FOV) dimensions (8 × 8 to 23 × 26 cm), voxel sizes (125 to 400 μm), presence of artifacts, spatial resolution, and degrees of MC cortication were selected.

For initially training the AI network, 40 CBCT scans were imported into Romexis software version 5.2.1.R (Planmeca, Helsinki, Finland) for tracing the MC using the built-in tool for nerve annotation. This initial training allowed the development of an initial version of a DL network capable of performing accurate voxel-wise MC segmentation, denoted as Virtual Patient Creator (Relu BV, Leuven, Belgium), which serves as a cloud-based AI tool. Then, 126 new CBCT scans were imported into this DL network to segment of the MC limits on cross-sectional slices by two oral radiologists. Afterward, these segmentations were doubled checked and used to train, refine, and robust the DL network. In addition, data augmentation strategies were applied to artificially increase the dataset and improve the generalizability and robustness of the model^21^.

**Figure 2:**
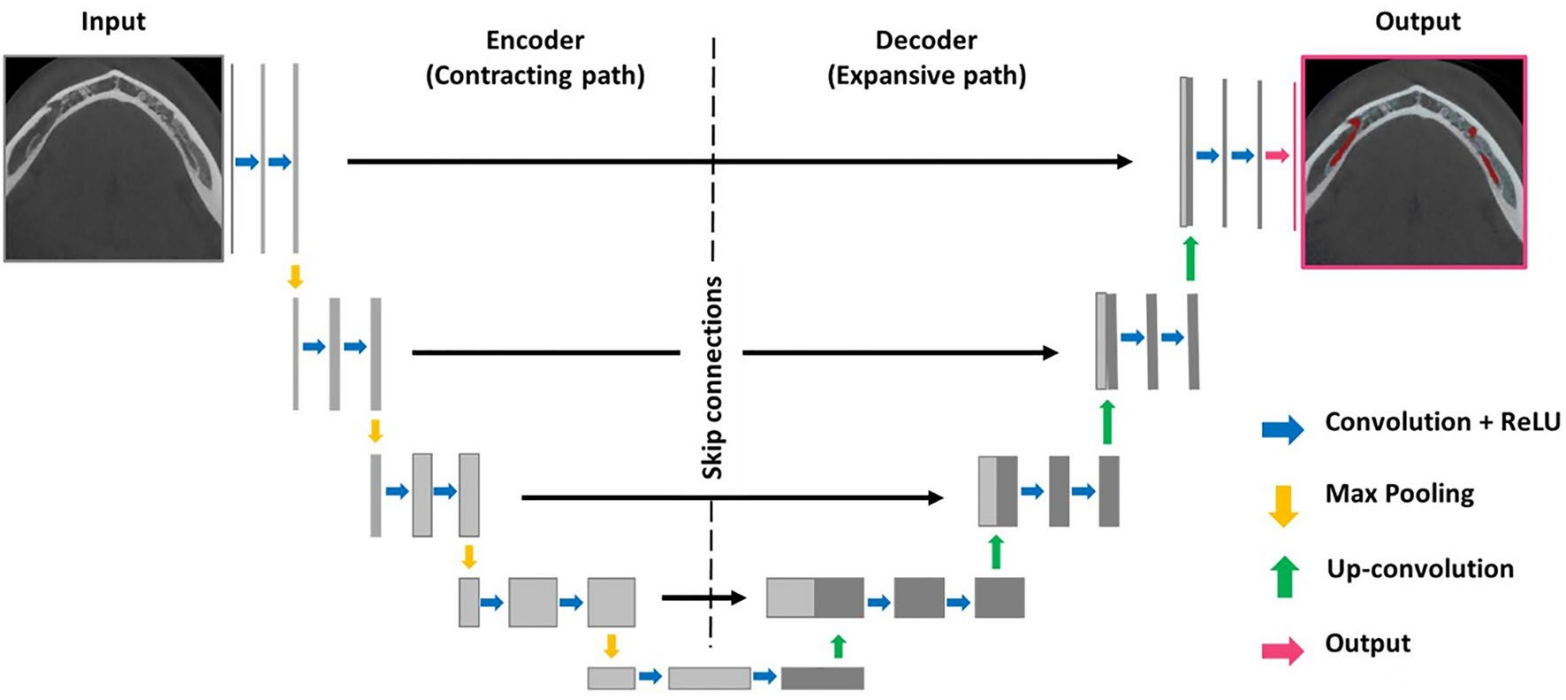
Representation of the 3D U-Net architecture. Adapted from Ronneberger et al.^26^.

The architecture to develop the MC segmentation output was based on multiple 3D U-Net networks, an encoder-decoder fully convolutional networks (FCN) with skip connections, applied in medical segmentation problems. The applied FCNs had two paths to allow better performance in classification and high-resolution segmentation of large images^21,26^. In the contracting path, the images were downsampled through convolutions, rectified linear unit (ReLU), and max pooling operations. Aiding to keep the border pixels lost in every convolution, skip connections propagated the context information to the symmetrical counterparts in the expansive path. Then, in the present study, the first path (encoder) performed a rough segmentation of the MC. The second path (decoder) refined the rough segmentation provided for the first path, producing a full-resolution MC segmentation. For this, the encoder extracted the features from the input image, creating a feature map with global information about the image. The decoder generated a dense segmentation mask of the input. Subsequently, skip connections were applied to combine the feature maps from the encoder to the decoder and improve the localization of the network. Then, a semantic segmentation combined the feature maps from all the layers of the decoder into one single output^26^ (Fig. 2).

### AI network optimization

For AI network optimization to detect AL, 93 new CBCT scans from the UZ Leuven Hospital M3BE database^31^ were collected for further network training, including 30 MC with AL. Therefore, a total of 219 CBCT scans were used for training of the network. The CBCT scans selected for the AI network optimization were from three different CBCT devices NewTom VGI EVO (QR Verona, Cefla, Verona, Italy), ProMax 3D MAX (Planmeca, Helsinki, Finland), and Accuitomo 170 (Morita, Kyoto, Japan), and they were acquired varying FOV dimensions (10 × 10, 12 × 8, and 15 × 12 cm), voxel sizes (160, 200, and 250 μm), mA (3–8), and kV (90 and 110). Besides this and to increase the robustness of the network, CBCT scans used in this step were from completely dentate or edentulous zone patients (age between 20–51 years old), varying the presence of artifacts and degrees of mandibular canal cortication, which could be fully corticated, partially corticated, or non-corticated. In addition, CBCT scans showing a FOV that not included both left and right MC and CBCT scans with movement artifacts that duplicated the MC path, were excluded from the sample.

**Figure 3:**
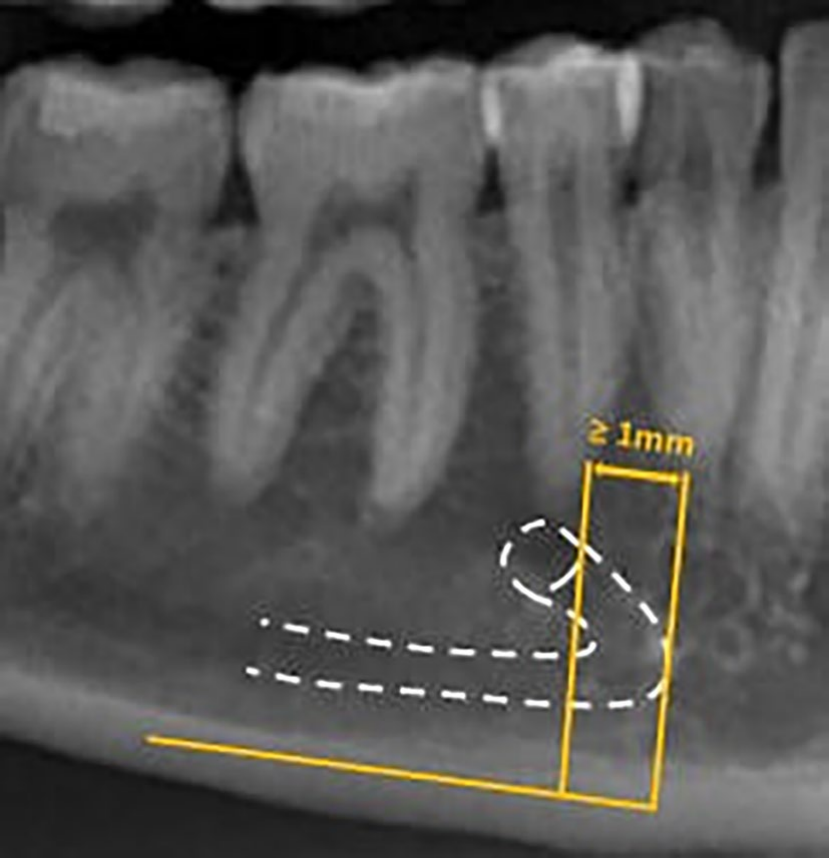
Schematic representation of the measurement of the anterior loop on a reconstructed panoramic image.

**Figure 4:**
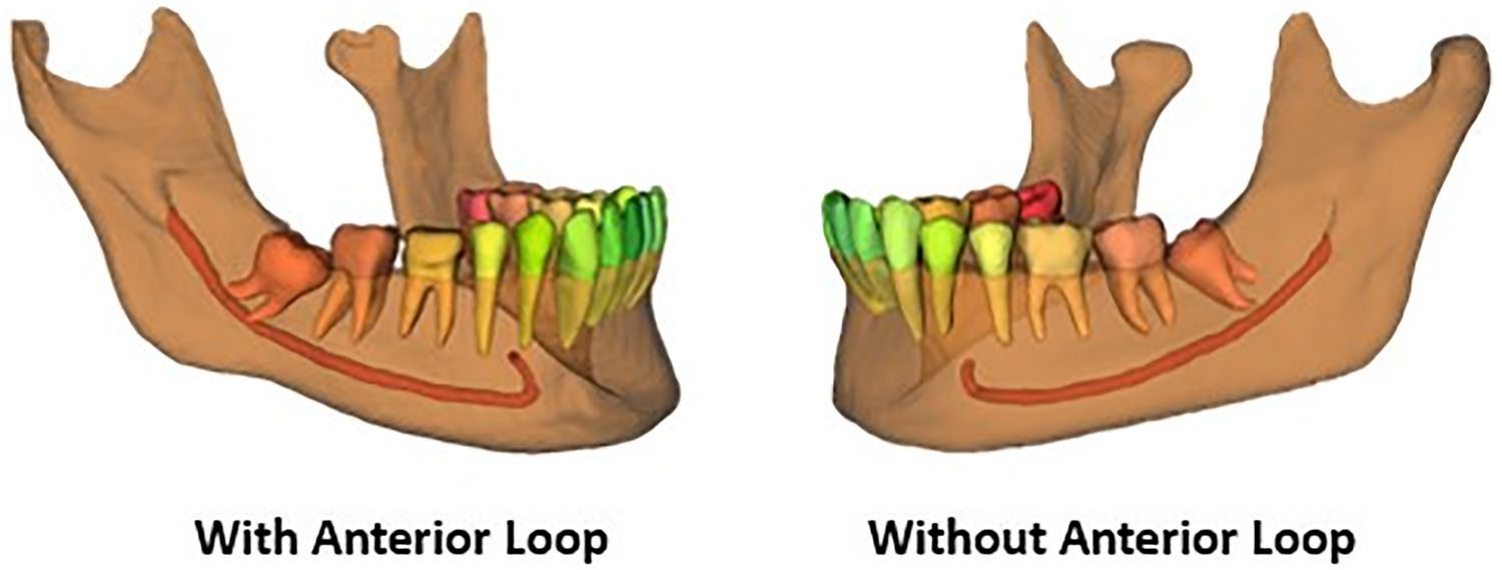
Representation of the segmentation of the mandibular canal with and without an anterior loop.

**Figure 5:**
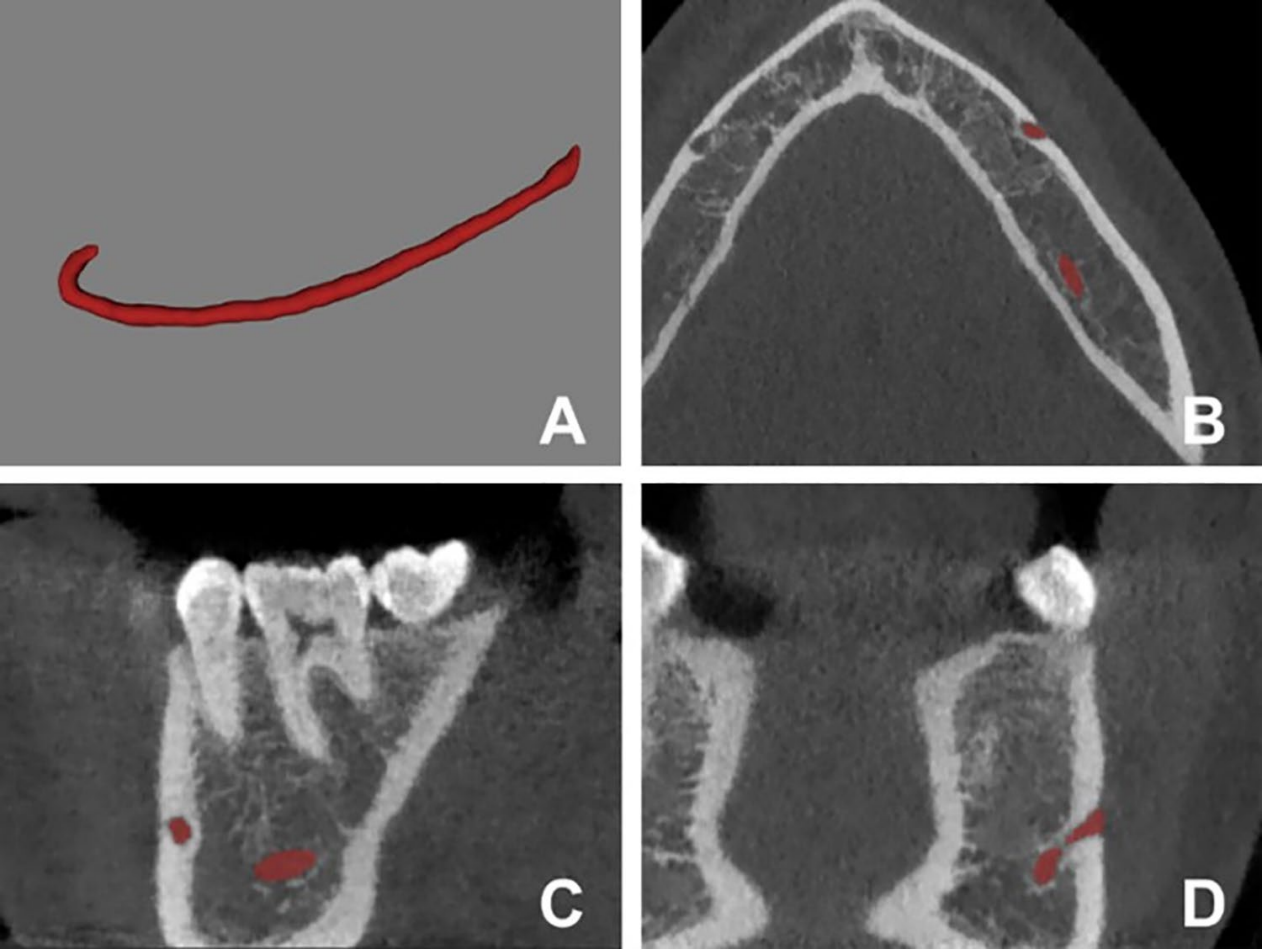
Mandibular canal segmented seen in a 3D view (**A**), axial view (**B**), sagittal view (**C**), and coronal view (**D**).

The presence of AL was established as described by Krishnan et al.^3^. It carried out assessing the horizontal distance between the tangent lines to the most anterior part of the loop and those to the anterior border of the mental foramen^3^. One radiologist measured this distance in a sagittal view oriented according to the mandible body. This measure was re-evaluated by another radiologist with more than five years of experience. Only cases of AL with ≥ 1 mm (Fig. 3) were considered^3^. AI generated MC segmentation of the cases (Fig. 4). Then two experts in oral radiology performed expert consensus segmentation, and if needed, they jointly adjusted manually the path, shape, and width of the canal as well as over- and underestimations on cross-sectional slices when deemed necessary (Fig. 5). During training, data augmentation techniques were applied to increase the dataset artificially. The techniques included random cropping and affine transformations (scaling, rotation, translation, shear, mirroring, and elastic deformations).

### Validation and Testing of the AI-optimized network

**Figure 6:**
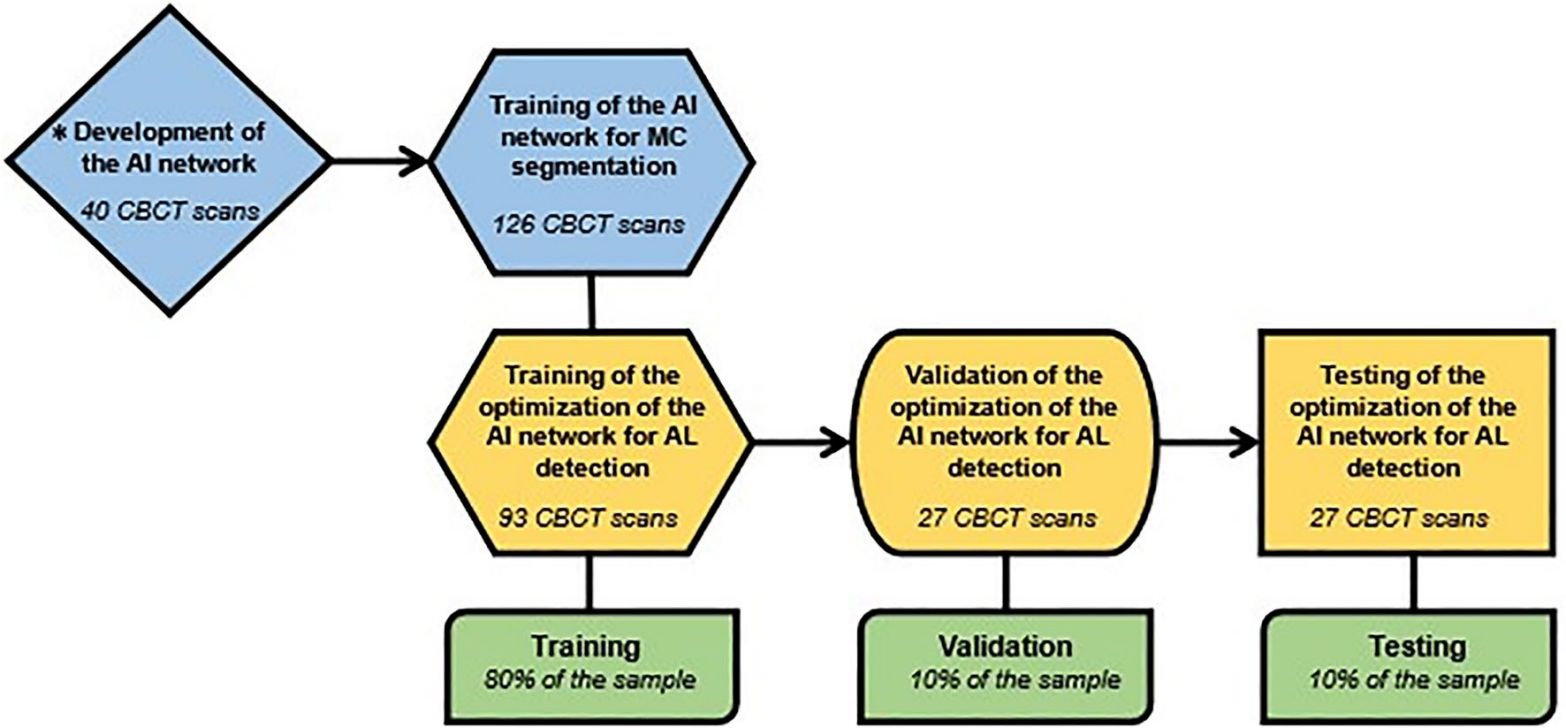
Flowchart of the sample distribution during the training, validation, and testing of the AI network. Blue forms were performed by Lahoud et al.^21^, while the yellow steps were performed in the present study.

**Figure 7:**
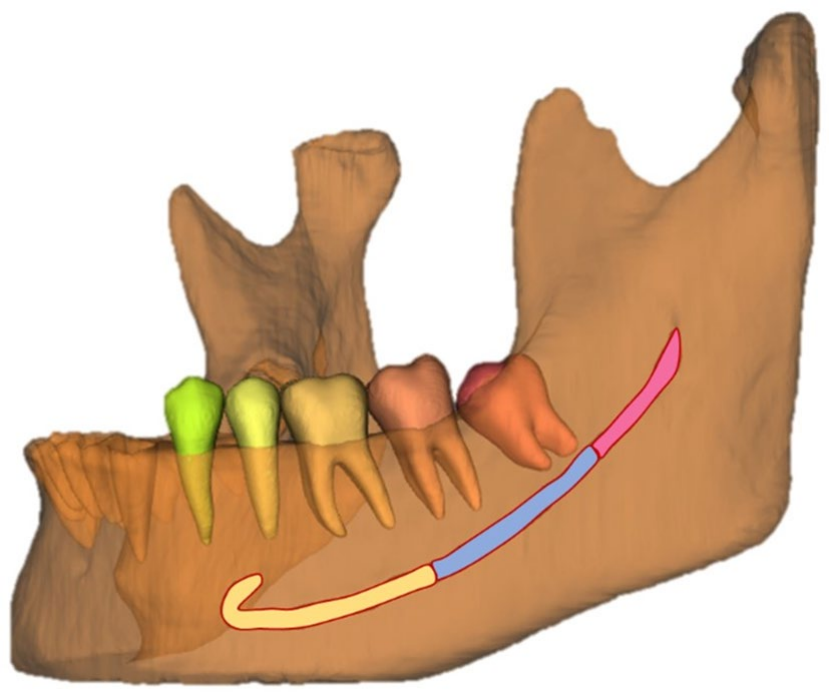
Mandibular canal divided into three sections: anterior (yellow), middle (blue), and posterior (pink).

Subsequently, 27 CBCT scans were used in the validation step to assess the performance of the AI-driven segmentation tool. Then, another 27 CBCT scans were used for the testing step (Fig. 6), in which 40 MC had AL, while 14 had no anatomical variation of the MC (control group). Subsequently, the MC was divided into three sections: anterior (region of premolars and first molar), middle (region of second and third molars), and posterior (posterior region to the third molar until the mandibular foramen) (Fig. 7). Then, the time to adjust AI-driven segmentation was recorded for each sections, primarily to allow a secondary analysis for the anterior section, that is crucial for surgery in the canine and premolar area, especially in the presence of AL. Furthermore, correct detection of the middle section is essential, e.g. for implant placement and third molar removal. Finally, the posterior section and the complete neurovascular canal segmentation are crucial for orthognathic surgery, trauma, and other maxillofacial surgical procedures.

The time spent to segment the MC automatically and to perform expert refinements of AI-driven over- and under-segmentation was recorded in each section. Segmentation of the AI-driven tool and its refinements were exported as Standard Tessellation Language files. The refined AI segmentation was considered the ground truth. Then voxel-level annotations were used to calculate the values of true positive (TP), false positive (FP), true negative (TN), and false negative (FN) regarding the number of pixels that the method predicted, as described below.

- TP: Voxels segmented by AI and segmented by the ground truth.
- FP: Voxels segmented by AI and non-segmented by the ground truth.
- TN: Voxels non-segmented by AI and non-segmented by the ground truth.
- FN: Voxels non-segmented by AI and segmented by the ground truth.

These values were applied to calculate the accuracy metrics for comparison of AI-driven and refined AI segmentation.

The following accuracy metrics were calculated:

- Intersection over union (IoU): Represents the similarity between the predicted object and the ground truth counterpart (area of overlap between expert and AI-driven segmentation that voxels match exactly). An IoU of 1 is a perfect segmentation^32^. It is defined by the equation:
  $$IoU = \frac{TP}{{FP + TP + FN}}$$
- Dice Similarity Coefficient (DSC): Amount of intersection between the AI-driven segmentation and the ground truth^33^, being a DSC of 1 considered as a perfect segmentation, defined by the equation:
  $$DSC = \frac{2 x TP}{{\left( {TP + FP} \right) + \left( {TP + FN} \right)}}$$
- 95% Hausdorff Distance (HDmm95): Indicates the 95th percentile of the largest segmentation error measuring the longest distance between a point in the ground truth (A) and its closest point in the AI-driven segmentation (B) measured in millimeters^34^. An HDmm95 of 0 is a perfect segmentation. Its equation is:
  $$\begin{aligned} 95HD\left( {A, B} \right) & = percentile \left[ {h\left( {A, B} \right) \cup h\left( {B, A} \right), 95th} \right] \\ h\left( {A, B} \right) & = max_{a \in A} min_{b \in B} \left\| {a - b} \right\| \\ h\left( {B, A} \right) & = max_{b \in B} min_{a \in A} \left\| {b - a} \right\| \\ \end{aligned}$$
- Precision: Based on region overlapping, measure the matching direction between the expert manual segmentation and the AI-driven segmentation, being the manual segmentation used as reference and 1 considered as a perfect segmentation^35^, defined by the following equation:
  $$Precision = \frac{TP}{{TP + FP}}$$
- Recall: Similar to the precision measure but using the AI-driven segmentation as reference. The Precision and Recall measure the agreement between the oriented boundary edge elements of the two segmentations^35^. A recall of 1 is considered a perfect segmentation. The recall equation is defined as:
  $$Recall = \frac{TP}{{TP + FN}}$$
- Accuracy: Weighted arithmetic mean that can be expressed both as weighted mean average of Precision and Inverse Precision and as weighted mean average of Recall and Inverse Recall^21^, with 1 being considered as perfect segmentation, defined by:
  $$Accuracy = \frac{TP + TN}{{TP + TN + FP + FN}}$$

Finally, the statistical analysis was performed using the GraphPad software (GraphPad Software, Inc., San Diego, CA, USA). The Mann–Whitney test was used to compare the accuracy metrics between the MC with and without AL. The Kruskal Wallis test compared the time of refinement between the MC with and without AL and between the MC sections (α = 5%).

## Data Availability

The datasets used and/or analyzed during the current study are available from the corresponding author upon reasonable request.
